# Cover crops lower the dispersal of grapevine foliar pathogens from the ground and contribute to early-season disease management

**DOI:** 10.3389/fpls.2024.1498848

**Published:** 2024-11-11

**Authors:** Gultekin Hasanaliyeva, Margherita Furiosi, Vittorio Rossi, Tito Caffi

**Affiliations:** ^1^ Department of Sustainable Crop Production (DI.PRO.VE.S.), Università Cattolica del Sacro Cuore, Piacenza, Italy; ^2^ Research Center for Plant Health Modelling (PHeM), Department of Sustainable Crop Production, Piacenza, Italy

**Keywords:** soil management, primary inoculum, air and splash dispersal, *Plasmopara viticola*, *Erysiphe necator*, disease development

## Abstract

Currently, fungicides are widely used to control grapevine foliar diseases. This study explored the possibility of decreasing the use of fungicides to control these diseases using cover crops in the inter-row of vineyards. In small-scale experiments, we found that cover crops (namely horseradish *Armoracia rusticana*) were able to (i) reduce the numbers of airborne conidia of *Botrytis cinerea* (originating from an inoculum source above the soil) escaping the cover canopy by >85% with respect to the base soil and (ii) reduce the number of raindrops impacting the soil by 46%–74%, depending on the cover crop height and rain-originated splash droplets that escaped from the ground by 75%–95%, which reduced splash-borne inoculum. In two organic vineyards, for 2 years, fall- (mixture of *Lolium perenne*, *Onobrychis viciifolia*, and *Trifolium repens*) or spring-sown (a mixture of *Vicia sativa* and *Sinapis* sp.) cover crops could significantly delay (by 14–30 days) and reduce (till >90%) the development of downy and powdery mildew epidemics. This effect was more evident in plots untreated with fungicides than in treated plots. Cover crops also delayed the onset of epidemics depending on the type of cover crop and disease. Cover crops did not negatively affect grape yield and quality. Overall, the results showed that the introduction of cover crops in vineyard management can significantly contribute to disease control by lowering the load from ground to grapevine canopies of pathogen inocula, delaying disease onset, and reducing diseases severity during the season.

## 1 Introduction

The viticultural sector is experiencing an increasing interest in organic viticulture, in which chemical fertilizers and synthetic pesticides are banned by Regulation CE 1584/2018 in the European Community (Commission Regulation (EC), 2018). Consumers and policymakers demand a reduction in chemical usage to limit negative impacts on human health and the environment (Carvalho, 2017), particularly regarding the contamination of terrestrial and aquatic ecosystems (Carvalho, 2017). It has been observed that bans on chemicals increase biodiversity by approximately 30% (Seufert, 2019), limiting the negative effects on positive organisms (Carvalho, 2017) but reducing grape yields by approximately 20%–25% (Seufert, 2019). In organic viticulture, disease management strongly relies on using copper- and sulfur-based fungicides that pose a threat to biodiversity (Pesce et al., 2024) and, to a lesser extent, on biocontrol microorganisms and chemicals of natural origin (La Torre et al., 2008; Provost and Pedneault, 2016; Jindo et al., 2022; Ortega et al., 2023). These latter solutions, however, often provide variable and insufficient disease control (Dagostin et al., 2011; Fedele et al., 2020; Caffi et al., 2022; Altieri et al., 2023).

To increase the efficiency of disease control, disease prediction models have been developed and implemented in decision support systems to identify infection periods and apply fungicides using a risk-based approach rather than based on calendar dates (Valdés-Gómez et al., 2017; Caffi and Rossi, 2018). Crop-adapted spray technologies have also been developed to define better the application dose of plant protection products and increase the quality of distribution (Gil et al., 2011; Planas et al., 2022). In recent years, there was an increasing interest in cover crops, i.e., plants sown into the vineyard (in the inter-rows and/or below the vine rows) to cover the soil for different purposes such as managing soil erosion, increasing soil fertility and health, improving water regulation, enhancing biodiversity and wildlife, and improving ecosystem functioning (Bettoni et al., 2016; Veiga et al., 2017; Garcia et al., 2018; Eckert et al., 2020; León et al., 2021; Abad et al., 2023). In addition, cover crops have been considered in the light of pest and disease control. A recent study showed that cover crops in vineyards slightly decreased the population of the European berry moth, *Lobesia botrana*, compared with traditional soil management (TSM) (Ranca et al., 2022). In fields, increased plant diversity leads to increased microorganism diversity and thus to increased beneficial effects against soil-borne pathogens (Larkin et al., 2010; Vukicevich et al., 2016; Garcia et al., 2018., León et al., 2021). For instance, *Brassica* species produce compounds that have fungitoxic effects against several soil-borne fungi (Hartwig and Ammon, 2002; Larkin et al., 2010; Vukicevich et al., 2016; Panth et al., 2020) and nematodes vectoring grapevine viruses (Aballay et al., 2004; Avato et al., 2013; Kruger et al., 2015). The potential role of cover crops in altering the dispersal of fungal spores from the ground to plants was also investigated (Ntahimpera et al., 1998; Hartwig and Ammon, 2002).

The function of cover crops in reducing the spore load from ground to vines may be of particular interest for viticulture because the soil serves as an inoculum source for several diseases. The primary inoculum of downy mildew (DM), caused by *Plasmopara viticola*, is represented by zoospores that disperse from the ground to vine canopies by rain splashes (Rossi and Caffi, 2012) after being released into water from the zoosporangia, produced by the oospores that overwinter in the leaf litter or the soil (Rossi et al., 2009; Maddalena et al., 2022). The primary inoculum of powdery mildew (PM), caused by *Erysiphe necator*, is composed of ascospores ejected by the chasmothecia that overwinter on vine bark (of trunks and cordons) and soil (Cortesi et al., 1997; Legler et al., 2014). Other pathogenic spores, such as *Botrytis cinerea* (the causal agent of gray mold), *Phyllosticta ampelicida* (black rot), *Coniella diplodiella* (white rot), and *Elsinoe ampelina* (anthracnosis) are produced by fruiting bodies on berry mummies in bunches have been infected in the previous year and retained on the trellis or the ground (Elmer and Michailides, 2007; Ji et al., 2021, 2023a, Szabó et al., 2023). In addition, the pathogens that cause grapevine trunk diseases can survive in the pruning material left on the ground and spread from this infected material (González-Domínguez et al., 2020; Kenfaoui et al., 2022; Ji et al., 2023b).

In this study, we (i) investigated the mechanisms through which cover crops can interfere with spore dispersal from the ground to vines, considering both air- and splash-borne dispersal paths, and (ii) compared the epidemics of DM and PM in organic vineyards whose inter-row soil was managed following the tradition or by sowing cover crop mixtures in fall or springtime. Some preliminary results were reported by Hasanaliyeva et al. (2021).

## 2 Materials and methods

### 2.1 Small-scale experiments

These experiments were conducted in small plots (25 m² in size, 5 × 5 m) to compare bare soil and cover crops when they were approximately 30, 60, and 90 cm in height. Plots with cover crops were sown with horseradish (*Armoracia rusticana*) at different times (i.e., at intervals of approximately 15–20 days) to obtain the above-mentioned soil coverage heights; *A. rusticana* is a perennial plant belonging to the *Brassicaceae* family, characterized by a large foliage area of up to 8000 cm^2^/plant before leaf senescence, a canopy of up to 120 cm in height, and great climatic adaptability; therefore, it can be sown in autumn and spring (De Maria et al., 2018; Rivelli and De Maria, 2019). The bare soil represented an undisturbed condition through which spores may spread without hindrance.

A first experiment (Exp 1) was conducted to evaluate the effect of cover crops on the dispersal of airborne conidia. An airflow was artificially created and directed across the plots using an electric fan, 50 cm in diameter (Korona Floor Fan 81004 – 145W, ltd, Korona Electric GmbH, Germany), placed on one side of each plot 20 cm above the soil. This fan was operated continuously for 24 h. Four Petri dishes with fresh-sporulating colonies of *B. cinerea* (approximately 10^6^ conidia per cm^2^ of colony) were placed horizontally on the ground in the middle of the plot—2 m from the fan—as an inoculum source. These dishes were prepared by growing *B. cinerea* conidia for 10 days on potato dextrose agar at 20°C under a 12-h photoperiod using white and near-UV (370 nm) light (Black Light UV-A, L18 w/73, OSRAM, Munich, Germany).

**Figure 1:**
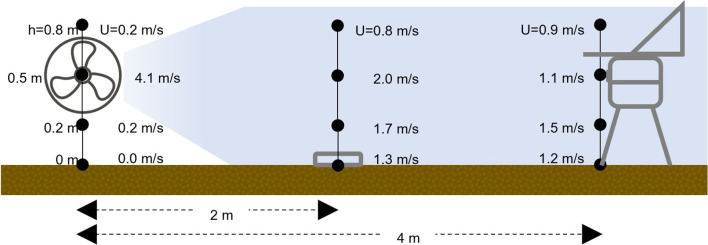
Schematic representation of plot setting for evaluating the effect of bare soil and cover crops on the dispersal of air-borne conidia (Exp 1). In the figure are represented: the fan (on the left) originating a continuous air flow, Petri dishes (in the center) containing sporulating colonies of *Botrytis cinerea* as inoculum sources, and the volumetric spore sampler (on the right) for capturing air-borne conidia; cover crops are not represented.

A volumetric spore trap (Lanzoni VPPS-2000, Bologna, Italy) was installed on the opposite side of the plot (opposite to the fan) to catch the *B. cinerea* conidia in the air current generated by the fan that crossed the plot (**Figure 1**). The spore sampler was operated for 24 h with a 220 V 50 Hz power source and adjusted to sample air at 10 L/min (Rossi et al., 2005), with the orifice set 50 cm above the ground. Afterward, the trapping surface (28 mm^2^/h wide) was removed and observed with the help of a microspore (675 × magnification) to enumerate the *B. cinerea* conidia, which were expressed as the number of conidia/m³ air per day. At 0.5 m above the ground), the fan generated a wind speed of 4.1 m/s on the bare soil, 2 m/s in the middle of the plot, where the Petri dishes with sporulating *B. cinerea* colonies were placed, and 1.1 m/s at the opposite side of the plot, where the spore trap was operated. This experiment was replicated three times on windless days (i.e., wind < 2 m/s).

A second experiment (Exp 2) was conducted to assess the effect of cover crops in reducing the number of raindrops impacting the ground, where inoculum sources of splash-borne pathogens are located. A rain simulator was installed 2.5 m above the plot’s ground level, which uniformly distributed 7 mm of rain during a single 150-second long rain event; raindrops generated by the rain simulator were different in size and had varying falling speeds. The rain simulator distributed “blue water,” which was prepared by dissolving an iron hydroxide powder in water (SIOF S.p.a.; Alessandria, Italy) (30 g of powder per liter of water). Twelve “impact-samplers” were placed randomly on the ground in each plot, consisting of strips of blotting paper (Gruppo Cordenons S.p.a., Milano, Italy) (12 × 12 cm) mounted on a wooden base so that raindrops impacting the sampler produced a blue imprint (Rossi and Caffi, 2012). Filter papers were removed after the rain event and were analyzed using an image analysis software (Assess 2.0; American Phytopathological Society Press, St. Paul, MN) to measure the area covered by blue drops (in cm²), which was expressed as the percentage of sampler area (i.e., 144 cm^2^). The experiment was performed with three replicates.

**Figure 2:**
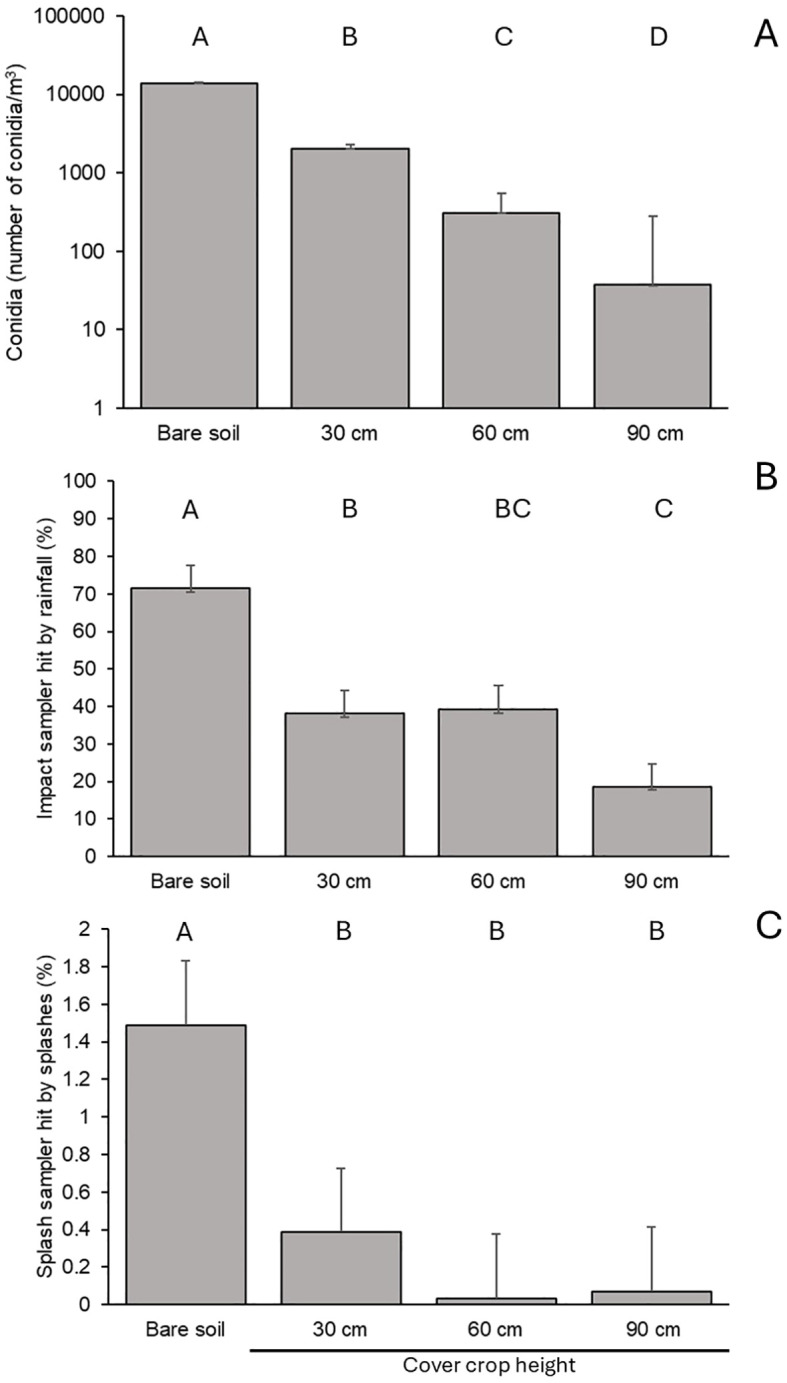
Effect of bare soil and horseradish cover crops of different height on numbers of *Botrytis cinerea* conidia caught by volumetric spore samplers in Exp 1 **(A)**, percentage of the surface of impact sampler placed on the ground covered by rain drops in Exp 2 **(B)**, and percentage of the surface of splash samplers positioned above the ground (average of samplers placed at 40, 80 and 140 cm above the ground) in Exp 3 **(C)**. Bars are averages and whiskers are standard errors; letters above the bars show significant difference at P=0.05.

A third experiment (Exp 3) was conducted to investigate the interference of cover crops on the splash droplets originating from the rain impacting the plot. The soil surface was uniformly covered with a blue layer (approximately 1 cm thick) of the iron hydroxide powder used in Exp 2 so that the splash droplets generated by the rain simulator were colored blue. The rain simulator was operated as described in Exp 2. Twelve “splash samplers” were installed in the plot to mimic grapevine leaves located 40 cm (i.e., the lower leaf layer), 80 cm (i.e., the bunch layer), and 140 cm (i.e., the higher leaf layer) above the soil (**Figure 2**); there were three samplers at each height level. The samplers were designed with a stick that supported a board (14 × 14 cm), which was fixed to the stick at an inclination of 45° and had two narrow slots that held a filter paper (12 × 12 cm) directed downward to simulate the abaxial surface of grape leaves. After a single rain event, the filter papers were removed and analyzed as described in Exp 2 until assessed as the percentage of sampler area occupied by splashing, blue droplets. This experiment was replicated three times, and the powder was re-applied after each simulated rain event to ensure uniform coverage of the soil.

### 2.2 In-vineyard experiments

Further experiments (Exp 4) were conducted in organic vineyards located in northern Italy in 2019 and 2020 at Castell’Arquato (Piacenza) and the University of Piacenza campus. Vineyards were planted with *Vitis Vinifera* cvs Croatina and Merlot, respectively, which are highly susceptible to diseases as grey mold, downy and powdery mildews; characteristics of the two experimental vineyards are shown in **Table 1**. Weather data were collected through automatic weather stations (Pessl Instrument GmbH, Weiz, Austria) installed in the vineyards. Cover crop mixtures were sown in the inter-rows of vineyards in fall (late October–early November) or in spring (late February–early March) in both years and compared with the TSM of the area, which was based on alternate inter-rows with bare soil and natural grassing. Cover crops were chopped and incorporated into the soil just before grapevine flowering. In both vineyards, the soil under each row was tilled mechanically. Fall-sown cover crop (FCC) was a mixture of *Lolium perenne* (48%), *Onobrychis viciifolia* (43%), and *Trifolium repens* (9%) at 35 kg/ha; spring-sown cover crop (SCC) was a mixture of *Vicia sativa* (92%) and *Sinapis* sp. (8%) at 65 kg/ha. Each of the three soil management conditions was applied to large plots, 0.5 ha in size, with three replicates; half the plots remained untreated (NT) throughout the season while the second half was treated (T) with copper- and sulfur-based fungicides according to information provided by a web-based decision support system vite.net^®^ (Horta s.r.l., Italy) (the applied products are listed in **Supplementary Material Table 1**).

**Table 1:** Characteristics of the two experimental vineyards, weather conditions between April and October, in 2019 and 2020.

Vineyard	Variety Rootstock	Year	Age (years)	Soil			Weather		
				Texture	pH	Organic matter (%)	Total rain (mm)	Rainy days (n)	Average temperature (°C)
Res Uvae (Castell’Arquato)	Croatina Kober 5BB	2019	20	silty-clay-loamy	6.9	1.3	835.2	72	19.7
		2020					460.4	69	19.8
University campus (Piacenza)	Merlot 420A	2019	15	loamy	7.0	0.9	519.5	80	20.3
		2020					438.6	69	20.3
40-year climate data^1^							483	65	19.1

^1^For University campus.

Starting from bud break, the plots were visited at least once per week to assess the incidence (as the percentage of symptomatic leaves/bunches) and severity (as the percentage of affected leaf or bunch area) of DM, PM, and gray mold on 100 random leaves and bunches from 10 contiguous plants (EPPO, 2002). The disease severity was used to calculate the area under the disease progress curve (AUDPC) following the method of Madden et al. (2007).

Vines growth stages were also defined according to Lorenz et al. (1995). Data on grape yield (kg/plant), berry sugar content (°Brix, using the refractometer RX-5000, ATAGO U.S.A., Inc.), pH (using the pH-meter CRISON GLP 22, Crison, Spain), and titratable acidity (g/L of tartaric acid equivalents, determined by titration with 0.1 N NaOH to a pH 8.2 endpoint) were collected at harvest from 30 random plants per plot.

### 2.3 Data analysis

An analysis of variance (ANOVA) was performed to test the effect of soil management types on spore dispersal data in small-scale experiments and for disease data in experiments conducted in vineyards; the number of conidia on spore samplers (Exp 1) was transformed using the natural logarithm function, and the percent of splash–sampler area covered by falling (Exp 2) or splashing (Exp 3) rain, as well as disease severity data (Exp 4) were transformed using the arcsin function before performing ANOVA. The Fisher Protected Least Square Difference test was used at P = 0.05 to separate the means.

Survival analysis was performed to analyze data on the first seasonal onset of DM and PM in vineyards. Survival analysis is a statistical approach that predicts event occurrence (“time-to-event”) (Westra et al., 1994; Scherm and Ojiambo, 2004)); in this case, the probability of disease onset. We compared the survival function using a log-rank test to check for the equality of survival functions for different soil management types and treated and untreated plots. A Cox proportional hazards regression model was used to assess the differences in disease onset between treatments, seasons, and locations. Models were compared using the likelihood ratio test. A P-value < 0.05 was considered significant. All statistical analyses were conducted using SPSS (ver. 29.0, SPSS Inc. Chicago, USA) software.

An ANOVA was also performed to test the effect of soil management type on the AUDPC of DM and PM on the leaves and bunches in vineyards, in which locations and years were considered as random variables. Since we were interested in the development of epidemics on both leaves and bunches, the AUDPC values on leaves and bunches were summed before analysis. The Student-Newman-Keuls test was used at P = 0.05 to separate means.

## 3 Results

### 3.1 Effects of cover crops on the dispersal of airborne spores

The spore traps in Exp 1 sampled 13960.0 (±241.9) *B. cinerea* conidia/m^3^ air per day on bare soil. The conidia in the air flowing across cover crops of 30, 60, and 90 cm in height were decreased significantly (P < 0.001) by 85.5%, 98.0%, and 99.7%, respectively (**Figure 2A**). The interaction between repeated experiments and soil management types was not significant (P = 0.987).

### 3.2 Effects of cover crops on the dispersal of splash-borne spores

The impact rain samplers located above the ground in the different plots (Exp 2) revealed significant (P < 0.001) differences between soil management types. In bare soil plots, 71.5% of the sampler area was impacted by raindrops, which was higher than in plots with cover crops of 30 and 60 cm in height (38.2% and 39.2%, respectively), and especially higher than the 90 cm tall cover crops (18.6%) (**Figure 2B**). Cover crops also significantly affected (P < 0.001) rain-originated splash droplets that escaped from the ground (Exp 3) (**Figure 2C**). On bare soil, the average sampler area covered by splash droplets was 1.4% for all sampler heights, which was higher than in all other treatments: 0.3% (on 30 cm tall cover crops), 0.03% (60 cm), and 0.07% (90 cm). Because of the significant (P = 0.003) interaction between soil management type and sampler height, the highest sampler area covered in splash droplets was approximately 3.8% in bare soil at the lowest sampler height (data not shown). Replicated experiments did not significantly influence the results of Exp 2 and Exp 3 (P > 0.15).

### 3.3 Effects of cover crops on disease epidemics in vineyards

In 2019, the grapevine growing season (April to October) experienced more precipitation than usual, especially in the Res Uvae vineyard, with the total precipitation being approximately 300 mm higher than the climatic average, while the 2020 growing season only experienced slightly more precipitation (**Table 1**). In both seasons, the total number of rainy days was higher than the average (**Table 1**). The average temperature of the two seasons was approximately 1°C higher than the climatic average, with higher temperatures at the University Campus than Res Uvae (**Table 1**). The grapevine phenology was similar across seasons and locations, with bud break occurring in the first half of April, full flowering between late May and the first decade of June, bunch closure between late June and early July, and veraison between the end of July and mid-August (**Table 2**). Weather conditions were favorable for the setting and development of the cover crop mixtures, with no evident prevalence by any species (data not shown).

**Table 2:** Dates of the main grapevine growth stages in the two Italian vineyards of **Table 1**, in 2019 and 2020.

Vineyard	Year	First leaf unfolded	Full flowering	Bunch closure	Veraison	Harvest
Res Uvae	2019	07/04	10/06	24/7	18/08	25/9
	2020	11/04	31/05	9/7	30/07	26/9
University campus	2019	31/03	10/06	18/7	14/08	27/9
	2020	13/04	26/05	4/7	22/07	18/9

**Figure 3:**
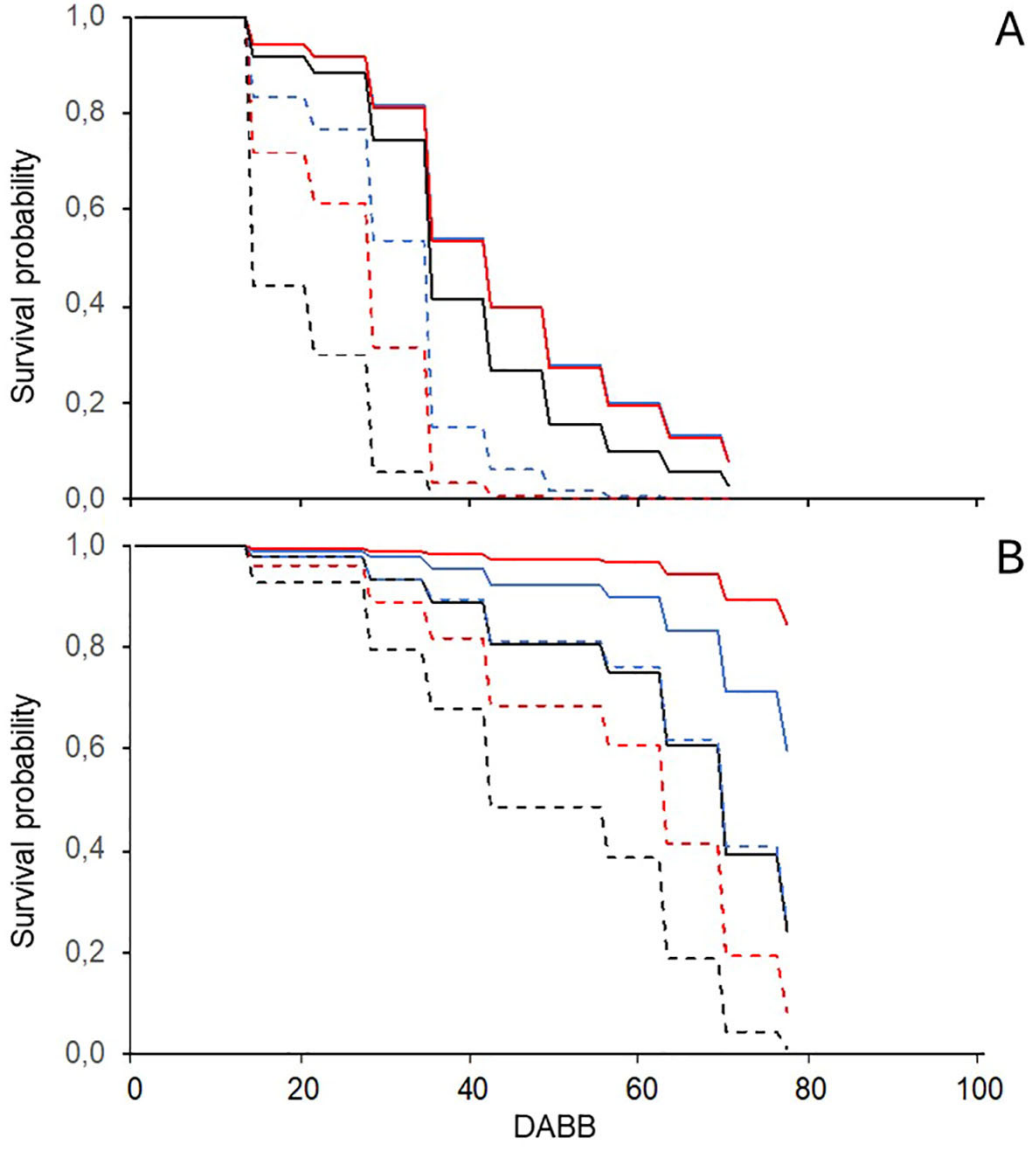
Probability functions for the time (as days after bud break, DABB) of first seasonal onset of downy **(A)** and powdery **(B)** mildew on leaves of grapevine plots that have been sprayed with fungicides (full lines) or left unsprayed (dotted lines) and with different soil managements types in the inter-row: traditional soil management (black lines; alternate inter-rows with bare soil and natural grassing), fall-sown cover crop (blue lines; a mixture of *Lolium perenne*, *Onobrychis viciifolia*, and *Trifolium repens*), and spring-sown cover crop (red lines; a mixture of *Vicia sativa* and *Sinapis* sp.).

DM and PM epidemics of differing severity developed in both seasons and locations, while gray mold did not. The survival analysis showed that the probability of DM and PM onset at different times during the season was affected significantly (P < 0.05 and P < 0.001 for the two diseases, respectively) by the soil management type and whether plots were treated. Considering a 50% probability of disease onset, DM was more likely to appear earlier in NT/TSM than in NT/SCC and NT/FCC, for which the survival functions estimated 14 and 21 day delays (**Figure 3A**). The disease onset in T/TSM is likely to occur at the same time as NT/FCC, while it is likely to occur 7 days later in T/FCC and T/SCC than T/TSM. PM was more likely to occur earlier in NT/TSM than in both NT/SCC and NT/FCC, with delays of approximately 20 and 30 days, respectively; in the T plots, PM never achieved a 50% onset probability (**Figure 3B**).

**Figure 4:**
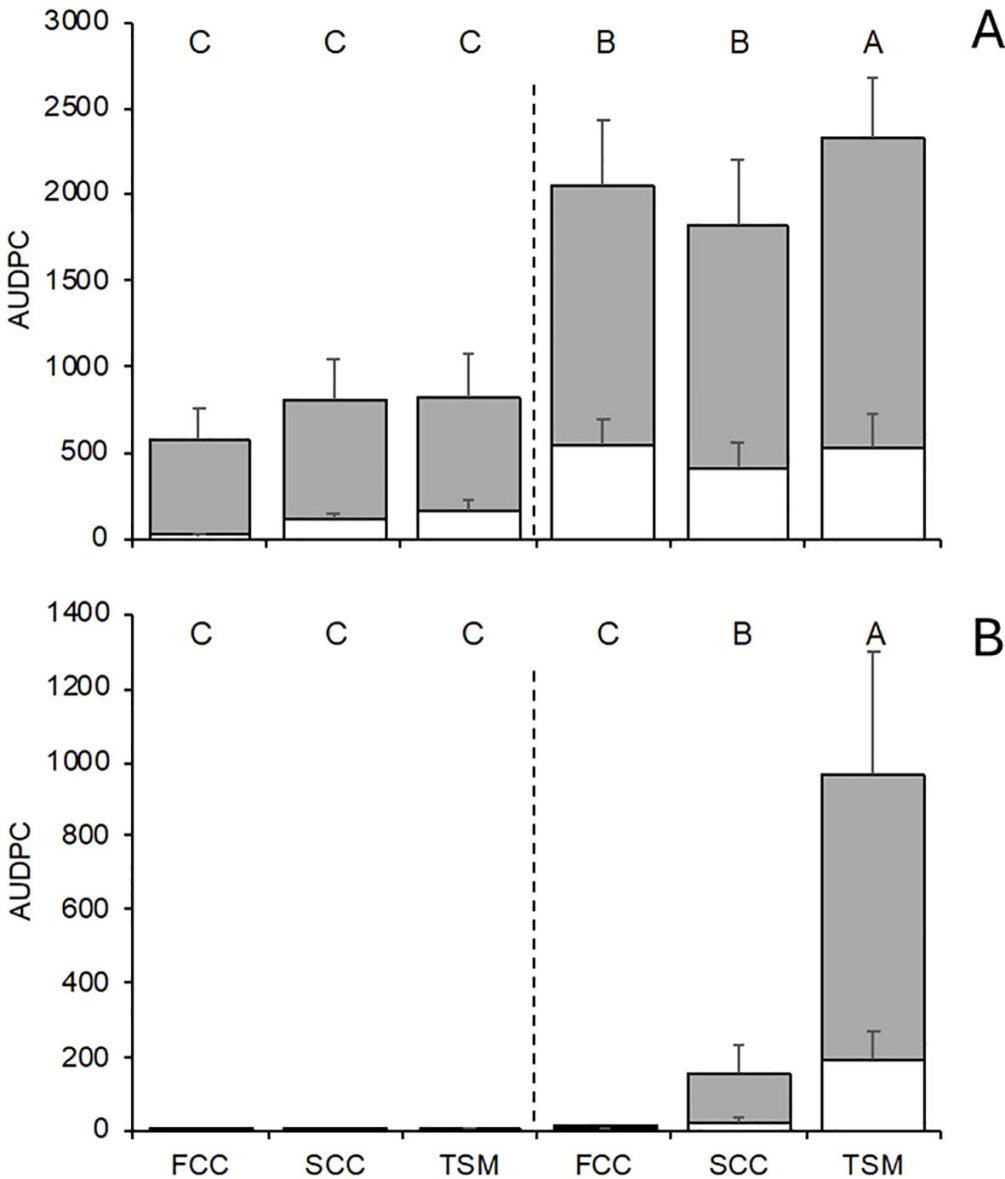
Area under the disease progress curve (AUDPC) of downy **(A)** and powdery **(B)** mildews on leaves (white bars) and bunches (grey bars) at the end of the season in grapevine plots treated with copper and sulfur fungicides (on the left of vertical line) and untreated (on the right), and with different inter-row soil managements: traditional soil management (TSM; alternate inter-rows with bare soil and natural grassing), fall-sown cover crop (FCC; a mixture of *Lolium perenne*, *Onobrychis viciifolia*, and *Trifolium repens*), and spring-sown cover crop (SCC; a mixture of *Vicia sativa* and *Sinapis* sp.). Bars represent the average of two vineyards and two years (see **Table 1**) and wishers are standard errors; letters above the bars show significant difference (at P=0.05) with reference to whole AUDPC (leaves and bunches).

Both DM and PM epidemics were influenced significantly by the interaction between location, season, and soil management type (P < 0.001). In NT, the average AUDPC for bunches was 3.1 times greater than for leaves for DM (**Figure 4A**), with average severities of 55.3% on leaves and 56.0% on bunches at the end of the season (not shown). PM also developed on bunches, with average severities of 16.1% on leaves and 16.3% on bunches in NT at season end (not shown); however, the AUDPC for bunches was 4.0 times greater than on leaves (**Figure 4B**). The soil management type did not show a significant effect on the overall DM AUDPC in treated plots; however, in NT the AUDPC was decreased significantly by 22.4% for SCC and 12.5% for FCC compared with TSM (**Figure 4A**). Similarly, there was no effect of the soil management type on the PM AUDPC in treated plots, while the AUDPC was reduced by 99.1% for SCC and 84.1% for FCC compared with TSM (**Figure 4B**).

Grape yield and technological parameters of berries were not affected by soil management practices (**Table 3**). The pH value was the only parameter showing a significant interaction between year and soil management practice (**Table 3**).

**Table 3:** Grape yield and technological parameters of berries in the two vineyards (average of 2019 and 2020), and results of ANOVA.

Vineyard	Soil management	Yield (kg/plant)	Sugar (°Brix)	pH	Tot acidity (g/l tartaric acid)
Res Uvae	Fall sowing	4.20	22.6	3.23	5.89
	Spring sowing	4.59	24.3	3.20	6.43
	Traditional soil management	5.28	23.9	3.21	6.53
University campus	Fall sowing	5.12	23.6	3.38	5.26
	Spring sowing	6.15	23.6	3.45	4.82
	Traditional soil management	6.57	23.6	3.38	5.93
Source of variability					
Year		ns	ns	0.016	ns
Soil management type		ns	ns	ns	ns
Vineyard × year		0.0001	ns	0.0085	ns
Year × soil management type		ns	ns	0.0041	ns
Vineyard × soil management type		ns	ns	ns	ns

NS, not significant.

## 4 Discussion

In the first part of this study, we evaluated the effect of cover crops on air- and splash-dispersal of fungal spores produced above the ground. In the second part, we evaluated the effect of cover crops on disease development in vineyards.

To evaluate the effect of cover crops on the dispersal of airborne spores, we used the conidia produced by *B. cinerea* on Petri dishes located above the ground, which mimic the conidia produced on berry mummies overwintered onto the vineyard ground. This study showed that cover crops significantly reduced both airflow across canopies and spore dispersal compared with bare soil and that this effect increased with canopy biomass—evaluated as the cover crop height—increased. The dispersal of airborne spores mainly depends on wind speed, which affects the number of spores transported and the distance they travel (Aylor, 1990; Mahaffee et al., 2023). When air currents cross plant canopies, the canopy biomass, structure, density, and flexibility all affect spore transport in air currents, by reducing the airflow across the foliage and by intercepting airborne spores that cannot escape the canopy (Legg, 1983; Ristaino et al., 1997; Aylor, 2017; Mahaffee et al., 2023).

Cover crops in the vineyard inter-row can reduce the number of airborne pathogen spores transported from the ground to grapevine foliage and bunches. In addition to *B. cinerea*, these mechanisms should work for other fungal pathogens. For instance, *Phaemoniella chlamydospora* produces conidiophores from hyphae and conidia (Crous and Gams, 2000), which are dispersed by air currents (Edwards and Pascoe, 2001, 2004; Moyo et al., 2014; Baloyi et al., 2016; González-Domínguez et al., 2020). *Phaeoacremonium* spp. also produce asexual spores in phialides (Mostert et al., 2006a, b) and form perithecia on decayed wood tissue (Rooney-Latham et al., 2005a; Bertsch et al., 2013; Baloyi et al., 2016), which discharge airborne ascospores in the presence of moisture (Rooney-Latham et al., 2005b). These fungi are causal agents of the Esca complex and may overwinter on affected canes retained above the vineyard ground. *E. necator* ascospores are also airborne after being discharged from chasmothecia overwintered above the soil or within the vine bark, even though the latter is prevalent (Rossi et al., 2010; Redl et al., 2021).

To evaluate the effect of cover crops on the dispersal of splash-borne spores from the ground, we measured both the amount of raindrops impacting the ground and the amount of splash droplets they produced. Compared with bare soil, cover crops significantly reduced the quantity of rain received by the ground surface and splash droplets transported to grape foliage, as well as the height these droplets traveled. Plant coverage of the soil reduces the amount of rainfall reaching the ground and the kinetic energy of the raindrops (Finney, 1984; Garcia et al., 2018; Novara et al., 2021). Rainfall intensity, in turn, affects the splash droplets produced by falling raindrops, including the number, size, speed, and trajectories of these splashed droplets (Fitt et al., 1989; Yang et al., 1992; Madden et al., 1996; Rossi and Caffi, 2012). Furthermore, the number of spores transported in splash droplets is proportional to droplet size: a higher drop diameter yields a higher number of spores (Fitt et al., 1988, 1989; Yang et al., 1992; Madden et al., 1996; Ristaino and Gumpertz, 2000). In addition, larger drops can reach higher heights during resplashes compared with smaller drops (Walklate et al., 1989; Rossi and Caffi, 2012).

The presence of cover crops in vineyard inter-rows can reduce the number of splash droplets and, therefore, spores reaching leaves compared with bare soil. Interestingly, cover crop height did not affect this effect; 30 cm high cover crops caused a significant reduction in rain splashes, as previously observed by Ntahimpera et al. (1998) for *Colletotrichum acutatum* conidia and Ahmed et al. (2002) for *Fusarium* ascospores. These experiments were conducted with horseradish, which formed a uniform layer of vegetation ground cover; however, the percent of soil coverage (Finney, 1984) and plant species (Thurow et al., 1987) may modulate the cover crop efficacy in reducing rain splashes, with perennials or shrubs being more effective than grasses.

Several grapevine pathogens produce primary inoculum from resting spores or fruiting bodies overwintering above the vineyard ground, which may be affected by cover crops. For instance, *P. viticola* overwinters as oospores in the leaf litter above the ground or in soil and repeatedly produces macrosporangia in the spring that release zoospores into water. These zoospores finally splash onto grape foliage via rain (Rossi et al., 2009; Rossi and Caffi, 2012). *Phyllosticta ampelicida*, the causal agent of grapevine black rot, can overwinter in fruit mummies buried or laid on the ground as perithecia, which later release ascospores in the presence of precipitation (Molitor and Beyer, 2014) or as conidia-containing pycnidia dispersed by rain splashes (Molitor and Beyer, 2014). *Coniella diplodiella*, the most common species causing grapevine white rot, overwinters as mycelium or pycnidia in mummified fruits, then pycnidia produces conidia that are dispersed to grapevine leaves mainly through rain splashes (Ji et al., 2024).

*Erysiphe necator* mainly overwinters as chasmothecia (Rossi et al., 2010; Redl et al., 2021). These fruiting bodies are produced in high numbers from late summer to leaf fall on fungal colonies powdering leaves and berries and are dispersed by rain splashes when mature (i.e., black in color) to the vine bark or ground (Rossi et al., 2010; Redl et al., 2021). Since only a small percentage of chasmothecia impacting the ground survive the winter (Rossi et al., 2010), it is commonly accepted that fruiting bodies retained on the bark represent the main source of primary inoculum in the spring (Rossi et al., 2010; Redl et al., 2021). Indeed, chasmothecia release ascospores when moistened by rain or prolonged humidity, which become airborne and cause primary infections (Rossi et al., 2010; Moyer et al., 2014); however, despite the fact that the number of chasmothecia impacting the ground may be significantly greater than those retained on the vine bark, even a small percentage that remain viable over the winter may contribute significantly to disease development in spring.

The results obtained in the first part of this study were confirmed in vineyards. The onset of both DM and PM was delayed by 2–4 weeks in cover crop plots compared with bare soil. In epidemiological terms, a reduction in the primary inoculum migrating to the plant—originating from cover crops in this case—causes a shift in the disease progress curve, as demonstrated by Vanderplank (1963) through his log-logistic equation. As a consequence, the disease epidemics—measured as AUDPC—were reduced significantly in untreated plots in which cover crops were sown in the spring (for DM) or in both the fall and spring (for PM) compared with bare soil or traditional management (alternating inter-rows with bare soil and natural grassing). This effect was not evident in treated plots, likely because of the low incidence of disease development in these plots.

Cover crops affected initial disease development, causing a reduction in both DM and PM epidemics. This was related to the polycyclic nature of mildew epidemics, in which a primary inoculum contributes to disease progress mainly from sprouting to flowering in both DM (Gobbin et al., 2005; Rossi et al., 2013) and PM (Calonnec et al., 2008; Rossi et al., 2010). Later on, secondary infections originated from the airborne sporangia of *P. viticola* (Gobbin et al., 2005; Rossi et al., 2013) and conidia of *E. necator* (Calonnec et al., 2008) produced by lesions on the grape foliage drive epidemic development. In addition, cover crops are usually cut before vine flowering to limit competition with grapevines at a time when nutrient and water demands are high (Celette and Gary, 2013; Garcia et al., 2018), thus ending their effects; however, leaving the cover crop foliage above the inter-row ground after cutting may create a mulching effect that reduces the dispersal of pathogens via splashing (Fitt et al., 1988; Madden and Ellis, 1990; Yang et al., 1990, 1992; Ristaino et al., 1997; Ristaino and Gumpertz, 2000). This mulching can also affect the dispersal of airborne spores originating from ground level, providing an obstacle for these spores to enter air currents (Teasdale et al., 2002).

The results showed an overall greater effect of fall sowing of cover crops compared with spring sowing. This may be related to the fact that cover crop establishment and development during the primary inoculum season (i.e., from sprouting to flowering) was higher in FCCs than in SSCs, so their effect on spore dispersal from the ground to vine canopies was higher. However, it should be considered that an increasing frequency of soil dryness in autumn due to climate change (Copernicus Emergency Management Service, 2024) can limit cover crop establishment (Schmitt et al., 2021) and, ultimately, their effect on spore dispersal the following spring. Caloiero et al. (2018) indicated that Mediterranean countries are the most prone to drought during fall, and Droulia and Charalampopoulos (2022) reported a reduction in average rainfall in most wine-producing European countries, especially in South France.

Overall, the results showed that cover crops can contribute to early-season disease management in vineyards, with no negative effects on grapevine yield and technological parameters. This can be considered an additional benefit of cover crops for plant health in viticultural systems. Indeed, diverse plant communities are more stable and balanced, with a higher presence of beneficial and natural enemies of arthropod pests (Liguori et al., 2011; Vukicevich et al., 2016; Garcia et al., 2018) and more abundant soil micro and macro-organism communities (Garcia et al., 2018) that can increase plant health and resilience through multiple mechanisms (van Bruggen et al., 2019). In addition, some cover crop species may act as vectors for mycorrhizal fungi (Cheng and Baumgartner, 2006), and others are known to produce volatile organic compounds that can attract or repel insect pests and their natural enemies (Hanna et al., 2003; Malone et al., 2022) or induce resistance toward pathogens in vines (Richards et al., 2020; BernasChina et al., 2023). Finally, some *Brassica* sp. release toxic compounds in soil, with a biofumigant effect against soil-borne pathogens (Guerra and Steenwerth, 2012; Vukicevich et al., 2016; Garcia et al., 2018) and virus-vectoring nematodes (Aballay et al., 2004; Avato et al., 2013; Kruger et al., 2015). Some cover crop plants, however, can be reservoirs or alternative hosts of grapevine pathogens. For instance, León et al. (2021) found that some cover crop species can host black foot disease pathogens (i.e., *Dactylonectria alcacerensis* and *Ilyonectria robusta*), such as *Plantago lanceolata* or *Pisum sativum*. Garcia et al. (2018) reported that some cover crop species, such as *Vicia sativa*, are alternative hosts for root-knot nematodes. These findings clearly indicate the importance of cover crop selection.

## 5 Conclusion

Overall, the results confirmed the potential benefits of cover crops as part of integrated pest management programs in vineyards. Indeed, cover crops may play a role in reducing the spore load from the vineyard ground to grape canopies for a number of both air- and splash-borne fungi (grey mold, downy and powdery mildew). This results in delayed disease onset in vineyards and reduced disease severity of mildew during the season. Further research is needed to identify the cover crop plants more suited to different viticultural environments and to define their optimal management strategy (from sowing to cutting and straw management) to obtain uniform soil coverage, dense biomass, and large-size plants in the early season (from vine sprouting to flowering) when most of the primary inoculum is mobilized from the ground to vine foliage. Further studies should also consider the long-term impact of cover crops on disease pressure in the vineyard and possible effects on yield and technological parameters of grapes.

## Data Availability

The raw data supporting the conclusions of this article will be made available by the authors, without undue reservation.
