# Supplementary material for: Cover crops lower the dispersal of grapevine foliar pathogens from the ground and contribute to early-season disease management
Source: Front Plant Sci. 2024 Nov 11;15:1498848. doi: 10.3389/fpls.2024.1498848 (PMC11586201; doi:10.3389/fpls.2024.1498848)
Supplement: Supplementary file 1 [file Table1.docx]

Supplementary Material

# Table 1. List of fungicides applied in the two vineyards (Res Uvae and University Campus) during seasons 2019 and 2020, application dates and dose applied (kg/ha).

| Vineyard | Date | Commercial Fungicide | Product Dose (kg/ha) |
| --- | --- | --- | --- |
| Res Uvae | 02/05/19 | Tiogold disperss | 3 |
|  |  | Verdram Hi Bio | 2,56 |
|  | 08/05/19 | Tiogold disperss | 2,56 |
|  |  | Verdram Hi Bio | 2,56 |
|  | 16/05/19 | Tiogold disperss | 3 |
|  |  | Verdram Hi Bio | 2,56 |
|  |  | Romeo | 0,28 |
|  | 23/05/19 | Tiogold disperss | 3,38 |
|  |  | Romeo | 0,19 |
|  |  | Verdram Hi Bio | 1,5 |
|  | 01/06/19 | Romeo | 0,28 |
|  |  | Tiogold disperss | 3 |
|  |  | Verdram Hi Bio | 0,75 |
|  |  | Champ DP | 0,75 |
|  | 09/07/19 | Champ DP | 1,88 |
|  |  | Verdram Hi Bio | 0,38 |
|  |  | Crittovit WG | 3,76 |
|  | 19/07/19 | Crittovit WG | 3,97 |
|  |  | Verdram Hi Bio | 1,58 |
| University Campus | 30/04/19 | Tiosol 80 WG | 0,4 |
|  |  | Airone più | 0,4 |
|  | 07/05/19 | Tiosol 80 WG | 0,8 |
|  |  | Airone più | 0,8 |
|  | 17/05/19 | Airone più | 0,8 |
|  |  | Tiosol 80 WG | 0,8 |
|  | 21/05/19 | Airone più | 0,8 |
|  |  | Tiosol 80 WG | 0,8 |
|  | 28/05/19 | Airone più | 0,8 |
|  |  | Tiosol 80 WG | 0,8 |
|  | 06/06/19 | Tiosol 80 WG | 2 |
|  |  | Airone più | 1,5 |
| Res Uvae | 17/04/2020 | Tiogold disperss | 2,1 |
|  |  | Cyprus 25 WG | 2,1 |
|  | 27/04/2020 | Tiogold disperss | 3,1 |
|  |  | Cyprus 25 WG | 1,56 |
|  | 08/05/2020 | Cupravit Bio Evolution | 1,09 |
|  |  | Tiogold disperss | 2,81 |
|  |  | Ibisco | 1,4 |
|  | 18/05/2020 | Ibisco | 2,73 |
|  |  | Verdram Hi Bio | 1,95 |
|  |  | Tiogold disperss | 3,9 |
|  | 03/06/2020 | Thiopron | 1,18 |
|  |  | Verdram Hi Bio | 1,56 |
|  |  | Ibisco | 1,95 |
|  | 15/06/2020 | Tiogold disperss | 4,67 |
|  |  | Verdram Hi Bio | 2,34 |
|  | 01/07/2020 | Verdram Hi Bio | 1,56 |
|  |  | Thiopron | 2,34 |
| University Campus | 25/04/2020 | Cuprofix C disperss | 1 |
|  |  | Tiogold disperss | 1 |
|  | 07/05/2020 | Cuprofix C disperss | 1 |
|  |  | Tiogold disperss | 1 |
|  | 15/05/2020 | Cuprofix C disperss | 1 |
|  |  | Tiogold disperss | 1 |
|  | 28/05/2020 | Cuprofix C disperss | 1 |
|  |  | Tiogold disperss | 1 |
|  | 08/06/2020 | Cuprofix C disperss | 1 |
|  |  | Tiogold disperss | 1 |
|  | 15/06/2020 | Cuprofix C disperss | 1 |
|  |  | Tiogold disperss | 1 |
|  | 02/07/2020 | Cuprofix C disperss | 1 |
|  |  | Tiogold disperss | 1 |
